# *Enterococcus faecalis* Bacteriophage 156 Is an Effective Biotechnological Tool for Reducing the Presence of Tyramine and Putrescine in an Experimental Cheese Model

**DOI:** 10.3389/fmicb.2019.00566

**Published:** 2019-03-20

**Authors:** Beatriz del Rio, Esther Sánchez-Llana, Begoña Redruello, Alfonso H. Magadan, María Fernández, Maria Cruz Martin, Victor Ladero, Miguel A. Alvarez

**Affiliations:** ^1^Department of Biotechnology of Dairy Products, Institute of Dairy Products of Asturias – Spanish National Research Council (IPLA-CSIC), Villaviciosa, Spain; ^2^Laboratoire Universitaire de Biodiversité et Ecologie Microbienne, Université de Bretagne Occidentale, Plouzané, France

**Keywords:** biogenic amines, tyramine, putrescine, biocontrol, dairy, *Enterococcus faecalis*, bacteriophage

## Abstract

Biogenic amines (BA) – nitrogenous compounds of low molecular weight – are the result of metabolism of certain amino acids. They are biologically present in all living organisms and play essential physiological roles. However, their accumulation in foodstuffs due to the metabolic activity of certain microorganisms represents a toxicological risk. Containing such microorganisms, and with an abundance of precursor substrate amino acids, fermented foods in general, and cheeses in particular, provide an ideal matrix for the accumulation of these toxic compounds. Unfortunately, the main microorganisms responsible for BA accumulation are members of the lactic acid bacteria (LAB) group, which are also essential for the development of the organoleptic characteristics of the final product. The methods used to reduce the BA content of cheese, such as milk pasteurization, commonly fail to do so, and affect desirable non-BA-producing LAB as well. Bacteriophages have been proposed as biotechnological tools for diminishing the presence of undesirable microorganisms in dairy products. Given their specificity, they could be used to target the population of BA-producing bacteria. In this work, we aimed to explore the use of *Enterococcus faecalis* infecting phages as a tool to reduce the content of BA in dairy products. For this, we proceeded to the isolation and characterization of *E. faecalis* bacteriophage 156, a member of the family *Myoviridae*. Its genome was sequenced and compared with that of *E. faecalis* family *Myoviridae* phages available in public databases. Its capacity to decrease the accumulation of the BA tyramine and putrescine in an experimental laboratory-scale cheese model was proven.

## Introduction

Biogenic amines (BA) are low molecular weight nitrogenous compounds – formed via the catabolism of certain amino acids – that posse important biological activities. However, these compounds can accumulate at high concentrations in foodstuffs due to the metabolic activity of certain microorganisms, and the ingestion of such foods can lead to intoxication (Ladero et al., 2010a). The presence of high concentrations of BA – mainly tyramine, histamine, and putrescine – in food constitutes a toxicological hazard (European Food Safety Authority [EFSA], 2011; del Rio et al., 2017, 2019).

Cheese has some of the highest detected BA concentrations (European Food Safety Authority [EFSA], 2011). Indeed, cheese provides a perfect environment for the production and accumulation of BA (Linares et al., 2012): (i) the milk used in cheese-making is not aseptic and contains microbial BA-producers that are part of the natural milk microbiota; (ii) the chemical and physical conditions of cheese-making (pH, temperature, etc.) favor the production of BA; and (iii) proteolysis plays an essential role in the ripening period that results in the release of large amounts of amino acids – the precursor substrates for BA production (Linares et al., 2012).

In cheese, and other fermented foods, the BA-producers are mainly some lactic acid bacteria (LAB); these form part of the normal microbiota of raw milk and participate in the fermentation process (Stratton et al., 1991; Spano et al., 2010; Linares et al., 2011). Unfortunately, in cheese production it is difficult to control BA accumulation even with Hazard analysis and critical control points (HACCP) systems implemented. Most treatments designed to do so, such as pasteurization and high pressure treatments, also have an impairing effect on other LAB species required for the correct elaboration of the fermented food (Novella-Rodriguez et al., 2004; Calzada et al., 2013). Further, these techniques are hardly ever fully effective (Novella-Rodriguez et al., 2002; Ladero et al., 2011). New methods targeting specifically BA-producing bacteria are consequently needed.

Tyramine and putrescine are the BA most frequently found in cheese at undesirably high concentrations (Fernandez et al., 2007; European Food Safety Authority [EFSA], 2011; Linares et al., 2011). The consumption of foods with elevated concentrations of these BA may result in hypertension, headache and migraine, etc. (Ladero et al., 2010a; Wunderlichova et al., 2014); susceptible people can be severely affected (McCabe-Sellers et al., 2006). Moreover, tyramine and putrescine have cytotoxic effect (Linares et al., 2016; del Rio et al., 2017, 2019), and in the case of the tyramine, it is even stronger than that of histamine, the solely BA for which a legal limit has been set, and even then only for some foods (European Food Safety Authority [EFSA], 2011). Furthermore, tyramine might be genotoxic for intestinal cells at concentrations easily found in BA-rich food (del Rio et al., 2018). Different bacteria isolated from the dairy environment can produce tyramine, putrescine or both BA (Linares et al., 2012; Romano et al., 2014; Ladero et al., 2015), but *Enterococcus faecalis*, which is widespread in raw milk, is that mainly responsible for tyramine accumulation in cheese. Together with certain strains of *Lactococcus lactis*, it is also largely responsible for putrescine accumulation (Ladero et al., 2010b, 2012a; O’Sullivan et al., 2015). Moreover, it has been shown that the production of tyramine and putrescine in *E. faecalis* is a species-level trait (Ladero et al., 2012b); developing strategies and biotechnological tools, e.g., *E. faecalis*-specific bacteriophages, that directly target its populations might therefore be of great use in the control of BA accumulation.

Bacteriophages are viruses that infect and kill bacteria. They have several interesting properties favoring their use as biocontrol agents in food matrices (Garcia et al., 2008; Mahony et al., 2011; Fernandez et al., 2017). They are innocuous to humans, animals and plants, can resist the environmental stresses encountered during food processing, and are relatively easy and cheap to isolate and propagate (Sillankorva et al., 2012). In addition, since they are self-replicating, only small quantities need be added to the matrix (Sillankorva et al., 2012; Ladero et al., 2016), and they have already been proposed as effective biotechnological tools for combating food-borne pathogens and spoilage bacteria (Garcia et al., 2008; Deasy et al., 2011; Yamaki et al., 2015; Ladero et al., 2016). Phages that infect specifically BA-producing microorganisms could be used as a new and precise tool to get rid of these dangerous bacteria, and consequently reduce the content of these toxic compounds in fermented foods, without having any side effect on other LAB members required for the fermentation process. However, these bacteriophages have a very constricted host range (Kot et al., 2014; Martínez et al., 2015), meaning that no single phage can likely serve as a biocontrol agent against different members of the LAB group. A cocktail of phages with different host ranges would have to be used (Chan et al., 2013). Phages from food matrixes able to infect strains of *E. faecalis* must therefore be identified, isolated, and typified to be able to apply them as biotechnological tools to reduce BA content in foods (Ladero et al., 2016).

In the present work, a bacteriophage, here named as *E. faecalis* bacteriophage 156, was isolated from cheese, its morphology, host range, and genome sequence, examined, its ability to reduce the *E. faecalis* population in a mini-cheese model determined, and the associated reductions in the final concentration of tyramine and putrescine evaluated. The results highlight the interesting qualities of this phage as a tool for controlling BA-producing *E. faecalis* populations, and thus improving dairy product safety and quality.

## Materials and Methods

### Bacterial Strains, Materials, and Media

A retail-purchased semi-hard cheese made from pasteurized ewe’s milk was used for bacteriophage screening. Different *E. faecalis* strains (18a, 19a, 23a, 28a, 63c, BA62, HFS 25, HFS59, CECT 4039, and CECT481^T^), were used as challenge hosts; the capacity of these strains to produce tyramine and putrescine has been reported (Ladero et al., 2009b, 2012b). To determine the host range of bacteriophage 156, additional *E. faecalis* strains were assayed (Table 1). To ensure that the phage do not infect technological relevant species, *L. lactis* (12 strains) and *Lactobacillus casei* (3 strains) strains were also tested (data not shown). All bacterial strains were grown in M17 broth (Oxoid, Spain) supplemented with 0.5% glucose (GM17) without aeration, except those of *L. casei* that were grown in MRS (Oxoid). In host strain assays, the culture medium was supplemented with 10 mM Mg_2_SO_4_ and 10 mM Ca(NO_3_)_2_ (MC-GM17 or MC-MRS). Phage titres were determined in double-layer agar plates, mixing 100 μl of serial dilutions in SM buffer (20 mM Tris–HCl pH 7.5, 1 mM Mg_2_SO_4_, 100 mM NaCl) with 100 μl of an overnight culture of the appropriate host strain. Plates were incubated at 37°C for 18 h and the resulting plaques counted. Unless otherwise stated, all reagents were purchased from Sigma-Aldrich (Spain).

**Table 1:** Host range of *E. faecalis* bacteriophage 156 (+ indicates strains sensitive to phage 156 infection).

Origin	*E. faecalis* strains	156 infection	Reference/collection
Type strain	CECT481^T^	+	CECT
Dairy	15a	+	Ladero et al., 2012b
Dairy	18a	+	Ladero et al., 2012b
Dairy	19a	+	Ladero et al., 2012b
Dairy	23a	+	Ladero et al., 2012b
Dairy	28a	+	Ladero et al., 2012b
Dairy	52c	+	Ladero et al., 2012b
Dairy	54c	+	Ladero et al., 2012b
Dairy	57c	+	Ladero et al., 2012b
Dairy	63c	+	Ladero et al., 2012b
Dairy	BA62	+	Ladero et al., 2012b
Dairy	BA64	+	Ladero et al., 2012b
Dairy	CECT 4039	-	CECT
Dairy	V61	+	Ladero et al., 2012b
Dairy	V63	+	Ladero et al., 2012b
Meat	LMG20645	-	LMG
Meat	LMG12161	+	LMG
Human	CECT795	+	CECT
Human	CECT4176	-	CECT
Human	HFS25	-	Ladero et al., 2009b
Human	HFS57	+	Ladero et al., 2009b
Human	HFS59	+	Ladero et al., 2009b
Human	HFS62	-	Ladero et al., 2009b
Human	HFS66	-	Ladero et al., 2009b
Human	HFS69	-	Ladero et al., 2009b
Clinical	JH2-2	+	Giard et al., 1996
Clinical	V583	+	Paulsen et al., 2003


*CECT, Colección Española de Cultivos Tipo; LMG, Laboratorium voor Microbiologie.*

### Biogenic Amine Determination

Biogenic amines were determined by ultra-high performance liquid chromatography (UHPLC). In broth they were quantified directly from a 100 μl sample, as described by Ladero et al. (2015). The BA in cheese samples were first extracted and quantified as previously described (Herrero-Fresno et al., 2012) with some modifications. Briefly, 1 g of cheese was mixed with 10 mL of 0.1 M HCl containing 0.2% (w/v) 3,3′thiodipropionic acid using an Ultra Turrax T50 homogeniser (OMNI International, United States) for 2 min at 20,000 rpm. The samples were then disrupted for 30 min in an ultrasonic bath and centrifuged at 5000 ×*g* for 30 min. After removing the fat layer, the supernatant was filtered through 0.45 μm cellulose acetate filters (VWR, Spain). The filtrates were deproteinised using ultra-filtration inserts (Amicon Ultracel-3K, Millipore) during centrifugation at 3500 *g* for about 1 h in a 5810 Eppendorf benchtop centrifuge (Eppendorf, Spain). Supernatant samples (100 μL) were then derivatised with diethyl ethoxymethylenemalonate as described by Redruello et al. (2013), and the BA separated out and quantified in an H-Class Acquity UPLC^TM^ UHPLC system (Waters, United States) running Empower 2 software (Waters), using the conditions described in the latter paper.

### Isolation of Phages From Cheese Samples

*Enterococcus faecalis* phage 156 was isolated from 1 g of sample cheese by enrichment culture and following the standard spot method in double-layer agar plates (Ladero et al., 2016). Enrichment cultures were inoculated with 100 μl of an overnight culture of host bacteria (*E. faecalis* strains 18a, 19a, 23a, 28a, 63c, BA62, HFS 25, HFS59, CECT 4039, and CECT481^T^; Table 1) and incubated without aeration at 37°C for 24 h. Samples were then placed in tubes and centrifuged (2000 ×*g* for 15 min) in a 5810 Eppendorf benchtop centrifuge, and 100 μl of the supernatant added to a new enrichment culture. After two rounds of enrichment, 10 μl were spotted onto double-layered agar MC-GM17 plates and incubated for 24 h at 37°C. When an inhibition halo was observed, the source supernatant was streaked to obtain single plaques. Some of these plaques were individually tested against the targeted host strains. For bacteriophage purification, a single plaque was picked up with a sterile tip, inoculated into 50 ml of MC-GM17 broth inoculated with the host strain, and incubated at 30°C until cell lysis was observed. The culture was then centrifuged (10,000 ×*g* for 15 min) in a 7780 centrifuge (Kubota, South Korea) with an AG6512C rotor, concentrated using the PEG/NaCl method (Binetti et al., 2005), and stored in SM buffer.

### Electron Microscopy

Concentrated phage particles were further purified in a continuous CsCl gradient by centrifugation (100,000 ×*g* for 20 h at 4°C) in an Optimax ultracentrifuge (Beckman Coulter, United States), as described by Ladero et al. (2016). Purified phage particles were stained with 2% uranyl acetate solution and electron micrographs produced using a CCD Gatan Erlangshen ES 1000 W camera coupled to a JEOL JEM 1011 transmission electron microscope (JEOL USA, Inc., United States) operated at 100 kV [performed at the Electron Microscopy Service of the Biotechnology National Centre (CNB-CSIC), Spain].

### DNA Isolation

Phage DNA was obtained from a concentrated suspension of phage particles following the procedure described by Binetti et al. (2005) as follows. Eighty microliters of lysis solution (0.25 M EDTA, pH 8.1; 0.5 M Tris–HCl, pH 9.6; 2.5% sodium dodecyl sulfate) were added to 400 μl of phage suspension and incubated in a water bath at 65°C for 30 min. One hundred microliters of 8 M potassium acetate were then added, and the mixture incubated on ice for 15 min and further centrifuged (16,000 ×*g*, 10 min, at 4°C) in a 5415R Eppendorf centrifuge. Phage DNA was precipitated from the supernatant with one volume of isopropanol, kept at room temperature for 5 min, and centrifuged again (16,000 ×*g*, 10 min at room temperature). The pellet was resuspended in TE buffer (10 mM Tris–HCl, 1 mM EDTA, pH 8.0) in the presence of 0.3 M sodium acetate pH 4.8, and precipitated twice with isopropanol for 5 min, followed by centrifugation (16,000 ×*g*, 10 min at room temperature). The DNA pellet was washed with 70% ethanol and absolute ethanol before being resuspended in TE buffer.

Microbial DNA from cheese samples was extracted from 1 g of cheese following the method described by Ladero et al. (2009a), which is based on the method of Ogier et al. (2002).

### Real-Time Quantitative PCR

The number of *E. faecalis* cells present in the cheese was measured by quantitative PCR (qPCR) using the specific primers tdcE4f and tdcE4r (Ladero et al., 2010b). Reactions were performed using the SYBR Green PCR Master Mix Kit (Applied Biosystems, United Kingdom) in 20 μl final volumes. For each reaction, 1 μl of template, 900 nM of each primer were added, along with 10 μl of SYBR Green PCR Master Mix containing ROX as a passive reference. Amplification and detection were performed using an ABI Prism Fast 7500 sequence detection system (Applied Biosystems) employing the standard program. The cycle threshold (Ct) values (automatically assigned by the thermocycler software) from 1/10 dilutions of cheese sample DNA were employed to calculate cell numbers using the equation cited by Ladero et al. (2010c).

### Phage Genome Sequencing

A genomic library of 0.5 kbp was constructed and subjected to 125 paired-end sequencing (providing approximately 800-fold coverage) using a HiSeq 1000 System sequencer (Illumina) at GATC Biotech (Germany). Quality filtered reads were assembled using SPADES software^1^ (Bankevich et al., 2012). Annotation was performed using the RAST server^2^ (Aziz et al., 2008), improving with results obtained from BLAST analysis^3^ (Altschul et al., 1997). The genome sequence was deposited at the European Nucleotide Archive (ENA) under accession number LR031359.

### Phylogenetic Analysis

A phylogenetic analysis of the *Myoviridae* *E. faecalis* bacteriophage sequences available in the NCBI database (Table 2) was performed by the Neighbor joining method after genome nucleotide sequence alignment undertaken using MAFFT v.7 software^4^ (Katoh et al., 2017). The tree was visualized in iTOL^5^ (Letunic and Bork, 2016).

**Table 2:** Characteristics of the phages subjected to phylogenetic analysis.

Phage	Family	Origin	Lifestyle	Genome size (bp)	Accession number
156	*Myoviridae*	Cheese	Lytic	141133	LR031359
ECP3	*Myoviridae*	Water	Lytic	145518	KJ801617
EF1	*Myoviridae*	–	Lytic	141996	MF001358
phiEF24C	*Myoviridae*	Water	Lytic	142072	AP009390
EFDG1	*Myoviridae*	Sewage	Lytic	147589	KP339049
EF5	*Myoviridae*	–	Lytic	141996	MF001361
EFLK1	*Myoviridae*	Sewage	Lytic	130952	KR049063
vB EfaP IME195	Podoviridae	Hospital sewage	Lytic	18607	NC_028693


### Biocontrol of BA Production in Cheese: Phage Challenge Assay

A BA biocontrol trial was performed in a laboratory scale Cabrales-type cheese model. Three replicates of cheeses were made following the procedure described by del Rio et al. (2016) with the following modifications. Milk was tyndallized by two steps of boiling during 15 min, letting it cool down slowly between the two steps until it reached room temperature. The milk was inoculated with *E. faecalis* 23a (10^4^ cfu ml^-1^), a dairy strain selected for its high capacity to produce tyramine and putrescine in cheese (Ladero et al., 2016), and divided in two batches. One of the batches was then challenged with bacteriophage 156 at a MOI of 0.1 (10^3^ pfu ml^-1^). Curd was supplemented with tyrosine (2 mM) and agmatine (5 mM), the precursors of tyrosine and putrescine, to ensure that substrate availability was not a limitation to maximum BA production. One sample from each of the three replicates was obtained at two steps; at the end of curd manufacture (i.e., just after whey draining and salting) (t0), and after 60 days of ripening at 12°C (tf). *E. faecalis* cells were then quantified by qPCR, phage particles by serial dilution and spotting onto double-layered agar MC-GM17 plates, and the tyramine and putrescine determined by UHPLC as indicated above.

### Statistical Analysis

Means ± standard deviations were calculated from at least three independent results, and compared using the Student’s *t*-test. Significance was set at *p* < 0.05.

## Results

### Bacteriophage Isolation

*Enterococcus faecalis* bacteriophage 156 (as it was thus named) was isolated from a semi-hard cheese, elaborated with pasteurized ewe’s milk, that had 0.08 mg kg^-1^ tyramine and 0.379 mg kg^-1^ putrescine. After cheese matrix homogenization in SM buffer, 100 μl samples were added to MC-GM17 cultures inoculated individually with the 10 *E. faecalis* host strains used for phage screening (Table 1). After two rounds of enrichment, in eight out of the ten *E. faecalis* strains tested was possible to observe a growth inhibition halo. These culture supernatants were then streaked to obtain isolated plaques. Three individual plaques picked from each of the analyzed plates were tested against all the *E. faecalis* strains used during the screening process. All showed the same phenotypically small-size and clear-halo plaque, infecting the same strains. It would therefore seem that a single phage was isolated. The *E. faecalis* type strain (CECT481^T^) was used to obtain from one isolated plaque a high titre bacteriophage stock for further characterization.

### Bacteriophage 156 Morphology and Host Range

To elucidate the phage 156 host range, 27 strains of *E. faecalis* from diverse sources (Table 1) were defied against the phage via the spot test. In addition, the phage was also tested against 15 strains of species with dairy technological importance such as *L. lactis* and *L. casei*. As expected, considering the usual narrow host range of LAB phages, none of the later strains was susceptible to the phage infection (data not shown). Twenty *E. faecalis* strains, out of the twenty-seven tested, of dairy, meat, human and clinical origin became infected (Table 1). In all positive cases, a clear halo was observed, indicating phage 156 to be lytic. It is noteworthy that phage 156 appears able to infect all but one of the *E. faecalis* strains of dairy origin tested.

**FIGURE 1:**
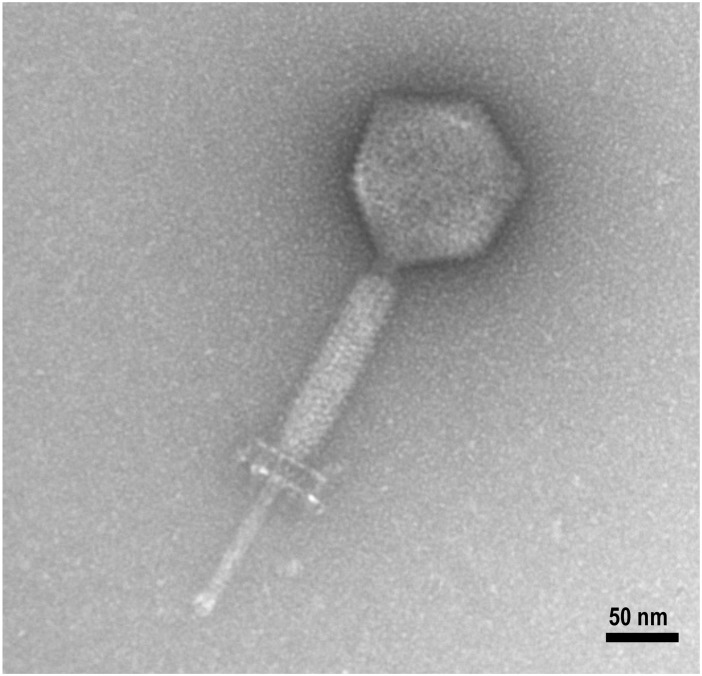
Electron photomicrograph of phage 156. Phage particles were prepared, negatively stained, and examined under the electron microscope as described in the Section “Materials and Methods.” The scale bar represents 50 nm.

Photomicrographs of phage 156 (Figure 1) revealed it to belong to the family *Myoviridae*. It had a short, rigid tail and isometric head, a small connecting neck between the head and tail, a decorated baseplate, and a large spike. The tail length was estimated at 109 ± 3 nm, the head diameter at 84 ± 3 nm, and the spike length at 68 ± 4 nm.

### Genetic Characterization of Bacteriophage 156

**FIGURE 2:**
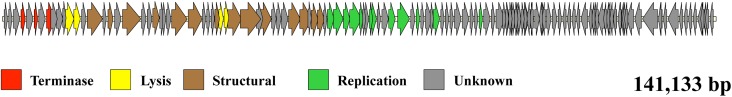
Phage 156 genome map. Diagram of the *E. faecalis* 156 phage genome. Each arrow represents an *orf* detected in the genome. The color code indicates the putative function assigned to each detected gene. The genome size (bp) is indicated.

To better characterize phage 156, and to make sure that it could be safely used as a biocontrol tool in food, its genome sequence was obtained. Total DNA was obtained and sequenced with Illumina technology at GATC Biotech. After assembly a contig representing the phage genome was obtained. The genome, which was 141,133 bp long, was annotated using the RAST server, and the annotation improved by BLAST comparison. It contained 209 open reading frames and 5 *tRNA* genes coding for sequences carrying the codons ACA, AGA TGG, ATC, and ATG (for threonine, arginine, tryptophan, leucine, and methionine, respectively) (Supplementary Table 1). The arrangement of the identified genes in the genome map revealed two regions that are divergently transcribed (Figure 2). Several genes relating to phage functions such as replication, packaging, capsid formation, and lysis were found, and grouped as functional modules (Figure 2 and Supplementary Table 1). However, most of the identified genes (>75%) showed no similarity with genes of known function and were annotated as hypothetical genes. Among them, none of the genes was identified as pathogenically related or toxin-encoding (Supplementary Table 1); phage 156 could therefore be safely used as a biotechnological tool in food, although before its application, safety trials should be performed.

**FIGURE 3:**
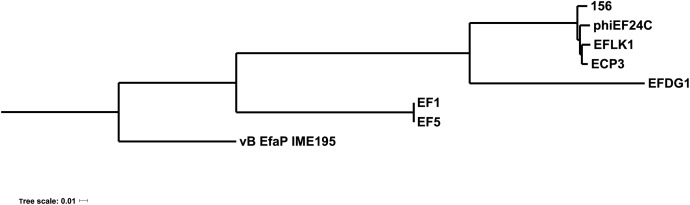
Phylogenetic tree of *E. faecalis* bacteriophages. Phylogenetic analysis of *Myoviridae* *E. faecalis* phage genomes available in databases. The name of each phage is shown (as represented in Table 2). The genome of the Podoviridae *E. faecium* phage vB_EfaP_IME_195 was included as a root.

The phage genome was then compared with the available genomes of other *Myoviridae* phages infecting *E. faecalis*. As shows in Figure 3, phage 156 appears especially closely related to phiEF24C, EFLK1, ECP3, and EFDG1. Some of these phages have been proposed as candidate tools for fighting enterococcal infections (Uchiyama et al., 2008; Khalifa et al., 2015, 2018).

### Phage-Based Tyramine and Putrescine Control in Cheese

**FIGURE 4:**
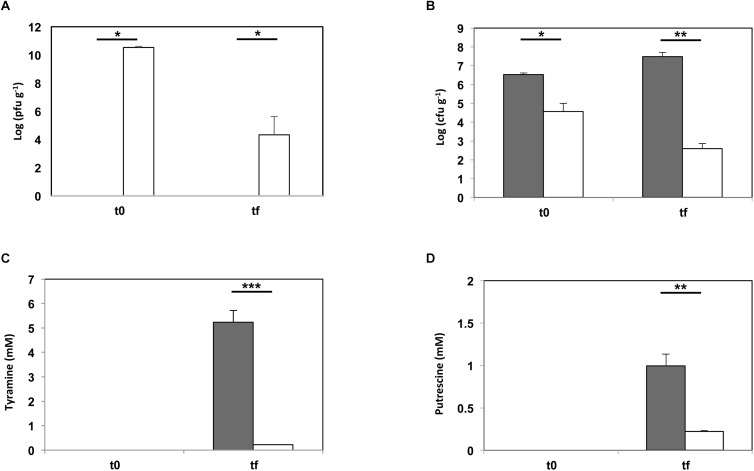
Phage biocontrol assay in a small-scale cheese model. *E. faecalis* 23a was challenged with phage 156 at an MOI of 0.1 (dark gray bar = control cheeses inoculated with *E. faecalis* 23a; white bar = cheeses inoculated with *E. faecalis* 23a and challenged with phage 156). **(A)** Number of phage 156 particles (log pfu gr^-1^) after manufacturing (t0) and after 60 days of ripening (tf). **(B)** Number of *E. faecalis* cells (log cfu gr^-1^) after manufacturing (t0) and after 60 days of ripening (tf), as calculated by qPCR. **(C)** Tyramine concentration (mM) measured by UPLC after manufacturing (t0) and after 60 days of ripening (tf). **(D)** Putrescine concentration (mM) measured by UHPLC after manufacturing (t0) and after 60 days of ripening (tf). An asterisk indicates a significant difference (^∗^*p* < 0.05; ^∗∗^*p* < 0.01; ^∗∗∗^ *p* < 0.001;Student’s *t*-test) with respect to control cheeses.

The ability of phage 156 to diminish the accumulation of putrescine and tyramine, by controlling the population of *E. faecalis*, was experimented in a cheese model assay. Samples were obtained at two steps at the end of curd manufacture (i.e., just after whey draining and salting) (t0), and after 60 days of ripening at 12°C (tf) (Figure 4). Virions were enumerated by serial dilution in double-layer agar plates (Figure 4A), *E. faecalis* cells were determined by qPCR (Figure 4B), and tyramine (Figure 4C) and putrescine (Figure 4D) concentrations by UHPLC, in both batches at both sampling times. In the batch challenged with phage 156, a large increase was seen in the number of phage particles at t0 (up to 3.3 × 10^10^ pfu g^-1^), indicating that the phage was able to propagate in the *E. faecalis* cells added to the milk during milk acidification and curd production (Figure 4A). The bacteriophage particles were much lower at the end of the ripening period (tf) than at t0, but phage particles (2.1 × 10^4^ pfu g^-1^) were still present and active (Figure 4A). The lytic proliferation of phage 156 significantly reduced the number of enterococci cells (t0, Figure 4B), even before ripening. At the final time sample (tf, Figure 4B), the drop in the number of enterococci cells was even greater (3.72 × 10^4^ versus 3.07 × 10^7^ cfu g^-1^; Figure 4B). Tyramine concentration in the cheese batches to which phage 156 had been added was reduced by more than 95% compared to controls (0.21 mM versus 5.24 mM) (Figure 4C). A significant reduction in putrescine was also observed when the phage was present (77.85%; 0.22 mM versus 0.99 mM for controls), although its reduction rate was lower than that reached by tyramine. However, the final concentration of putrescine accumulated was also lower than that of tyramine. In summary, the occurrence of bacteriophage 156 effectively reduced the accumulation of both tyramine and putrescine.

## Discussion

It was characterized a new lytic bacteriophage – named *E. faecalis* bacteriophage 156 – isolated from a cheese made from pasteurized ewe’s milk, which was found able to infect *E. faecalis*, one of the principal tyramine and putrescine producers in raw milk cheeses (Ladero et al., 2010b, 2012a; O’Sullivan et al., 2015). Thus, its capability to reduce the cheese content of tyramine and putrescine was further evaluated in an experimental cheese model assay.

According to the observed morphology, phage 156 belongs to *Myoviridae* family (Figure 1). Most of *Myoviridae* LAB-infecting virus isolated to date have been described as forming a low heterogenicity group in opposite to bacteriophages of other families like *Siphoviridae*, to which the majority of LAB-phages isolated so far belong (Mahony and van Sinderen, 2014). Phage 156 infected almost 75% (20/27) of the tested *E. faecalis* strains, including many of different origin (Table 1). This might be then considered a broad host range for a dairy phage, since most of the isolated virus infecting LAB strains show a very constrained range (sometimes just one single host strain) (Kot et al., 2014; Martínez et al., 2015). This present range is certainly above average when compared with those described in the literature (Lee et al., 2014). Nevertheless, *E. faecalis*-infecting phages of the family *Myoviridae* tend to possess broader host ranges than do *Siphoviridae* family members (Uchiyama et al., 2008; Lee et al., 2014).

The phage 156 genome was 141,133 bp long, i.e., within the range (130–147 kbp) of other *Myoviridae* *E. faecalis* phages (Table 2). A total of 209 *orf* and 5 *tRNA* genes were detected (Supplementary Table 1). In a previous comparison among *Myoviridae* *E. faecalis* phage genomes (Khalifa et al., 2018), it was reported that the most remarkable differences resided in the number of *tRNA* genes present, ranging from 0 in the EFLK1 genome (Accession number KR049063) to 24 in the EFDG1 genome (Accession number KP339049). The phage 156 genome has 5 *tRNA* genes located in a position similar to that seen for phiEF24C and ECP3; the EF1 and EF5 phage genomes contain 10 *tRNA* genes. Only the *tRNA* gene for the sequence carrying codon AGA (for arginine) is common to all the analyzed genomes; the other *tRNA* genes present showed great diversity. The presence of *tRNA* genes in some phages has been related to different codon usage, resulting in improved phage protein production compared to the host, differences in infection efficiency at different phases of bacterial host growth, and differences in burst size. However, in the case of *Myoviridae* *E. faecalis* phages, the roles of the different *tRNA* gene numbers and their diversity remain unclear (Khalifa et al., 2018). Phage 156 is closely related to other *Myoviridae* *E. faecalis* phages for which genomes are available in the literature (Figure 3). The phylogenetic tree constructed in this work (Figure 3) incorporates three new genomes (156, EF1 and EF5) over that previously reported by Khalifa et al. (2018). Those of phages EF1 and EF5 seem to form a separate group. Interestingly, phage 156 grouped with other phages that have been described as potential tools for combating *E. faecalis* infections (Uchiyama et al., 2008; Khalifa et al., 2018).

Phage 156 was shown to strongly reduce the accumulation of tyramine and putrescine in cheese, an effect clearly mediated by the reduction caused in the population of *E. faecalis* cells. *E. faecalis* is the predominant enterococcal species found in raw milk, reaching titres of 10^2^–10^3^ cfu ml^-1^ (McAuley et al., 2015). Moreover, *E. faecalis* is the principal microorganism responsible for the accumulation at high concentrations of both tyramine and putrescine in cheese (Ladero et al., 2012a; O’Sullivan et al., 2015). The capacity to produce both BA has been described as a species-trait characteristic in *E. faecalis* (Ladero et al., 2012b).

In this work, 10^4^ cfu ml^-1^ of a dairy origin *E. faecalis* strain were inoculated as a starting concentration for the cheese model. This concentration has been established as the threshold at which the risk of accumulating BA at high concentrations begins (Ladero et al., 2008, 2010c). *E. faecalis* was then defied with 156-phage at a MOI of 0.1 (10^3^ pfu ml^-1^). As shown in Figure 4A, the phage was able to proliferate during the first stages of cheese production, curd formation and salting. During these initial steps, the *E. faecalis* population also grew (by two orders of log magnitude) (Figure 4C), while in the presence of bacteriophage 156 no growth was observed. The finding that such a small titre of virions was capable to prevent the multiplying of *E. faecalis* (Figure 4B) shows that adding it to raw milk might efficiently decrease *E. faecalis* numbers in cheese products. As expected, the reduction in the *E. faecalis* count resulted in a concomitant reduction in the concentration of both tyramine and putrescine. Indeed, the reduction in the tyramine concentration was >95%. In the control cheese it reached a concentration of 5.24 mM, equivalent to 718.8 mg of tyramine per kg^-1^ of cheese, a value that clearly exceeds the suggested safety limit (200–500 mg kg^-1^) (Alvarez and Moreno-Arribas, 2014; Linares et al., 2016). In other hand, in the cheese in which the phage was present, tyramine concentration was held to 29.13 mg kg^-1^, far below this limit. The observed reduction in the tyramine concentration was even higher than that observed in a similar experiment involving *E. faecalis* phage Q69 – a *Siphoviridae* family phage – in which an 85% reduction in the tyramine concentration was observed (Ladero et al., 2016). In both cases, the addition of phage particles to the milk resulted in cheeses with a safe tyramine content. Moreover, in the present work, a 77.8% reduction in the putrescine concentration was observed.

Interestingly, at the end of the ripening period, phage particles were still detected in relatively high numbers (10^4^ pfu ml^-1^). The presence of active phage particles at this point may help to avoid the accumulation of tyramine and putrescine during storage, a critical period in which BA-producing enterococci might increase in number if the storage temperature is inadequate (Martinez et al., 2011). Food-borne spoilage microorganisms can reach very high numbers if handling and storage conditions are inadequate; this has a negative impact on food quality and therefore on its economic value. However, if the population of spoilage microorganisms remains under control, their effects could be kept to tolerable limits (i.e., the occurrence of BA beneath the suggested safety threshold).

In summary, in the present work we report the isolation – from the dairy environment – and characterization of *E. faecalis* phage 156. This phage efficiently reduced the final concentration of tyramine and putrescine in an experimental cheese model. In real cheese manufacture, however, the number of *E. faecalis* strains present, either in the raw milk or appearing as later contaminants of the cheese matrix, would almost certainly be higher. The idea that a single phage, even with a broad host range, might be able to infect them all is too hopeful. So, a cocktail of well characterized phages with different host ranges could solve this problem (Nobrega et al., 2015). Our group has previously shown the effectiveness of a *Siphoviridae* phage in reducing the accumulation of the BA tyramine in an experimental cheese model (Ladero et al., 2016); the present work provides an additional resource that might help achieve this objective. In the light of the obtained results, and given the fact that *E. faecalis* is responsible for most of the tyramine and putrescine accumulation in cheese, we propose that a bacteriophage biocontrol strategy be followed during the manufacture and storage of this food. This should allow for the production of safer fermented dairy products.

## Author Contributions

BdR and VL designed and carried out some experiments and drafted the manuscript. AM performed the phage genome analysis. ES-L and BR performed some experiments. BdR, MM, and MF participated in the design of the study and helped to write the manuscript. MA provided the general concept and supervised the work and the writing of the manuscript. All authors contributed to the discussions surrounding the work and approved the final version of the manuscript.

## Conflict of Interest Statement

The authors declare that the research was conducted in the absence of any commercial or financial relationships that could be construed as a potential conflict of interest.

## Footnotes

1 http://cab.spbu.ru/software/spades/

2 http://rast.nmpdr.org/rast.cgi

3 http://blast.ncbi.nlm.nih.gov

4 https://mafft.cbrc.jp/alignment/server/

5 https://itol.embl.de/
