# Supplementary material for: Enterococcus faecalis Bacteriophage 156 Is an Effective Biotechnological Tool for Reducing the Presence of Tyramine and Putrescine in an Experimental Cheese Model
Source: Front Microbiol. 2019 Mar 20;10:566. doi: 10.3389/fmicb.2019.00566 (PMC6435515; doi:10.3389/fmicb.2019.00566)
Supplement: Supplementary file 1 [file Table_1.DOCX]

Supplementary Material

***Enterococcus faecalis* bacteriophage 156 is an effective biotechnological tool for reducing the presence of tyramine and putrescine in an experimental cheese model**

Beatriz del Rio, Esther Sánchez-Llana, Begoña Redruello, Alfonso H. Magadan, María Fernández, Maria Cruz Martin, Victor Ladero*, and Miguel A. Alvarez

Department of Technology and Biotechnology of Dairy Products, Dairy Research Institute, IPLA-CSIC, Villaviciosa, Spain.

*** Correspondence**: ladero@ipla.csic.es

**Supplementary Table 1:** Features of *Enterococcus faecalis* bacteriophage 156. The *Orfs*, gene number, and gene position in the phage 156 genome are shown, as are the predicted functions, molecular weights and isoelectric points of the encoded products. The top BLAST hit and E-value are also indicated.

| Orf | Gene | Start (bp) | Stop (bp) | Length (aa) | MW (kDa) | pI | Predicted function | BLAST_HIT | E-VALUE |
| --- | --- | --- | --- | --- | --- | --- | --- | --- | --- |
| 001 | phi156_001 | 47 | 316 | 89 | 10.10 | 9.84 |  | *Enterococcus* phage phiEF24C_NC_009904: hypothetical protein; | 2.08E-57 |
| 002 | phi156_002 | 337 | 615 | 92 | 10.28 | 9.87 |  | *Enterococcus* phage phiEF24C_NC_009904: hypothetical protein; | 7.04E-59 |
| 003 | phi156_003 | 619 | 1047 | 142 | 16.17 | 4.39 |  | *Enterococcus* phage EFLK1_ORF203; hypothetical protein; | 1.74E-95 |
| 004 | phi156_004 | 1047 | 1415 | 122 | 13.82 | 9.59 | Phage terminase, Large subunit | *Enterococcus* phage phiEF24C_NC_009904: putative large terminase; | 3.25E-88 |
| 005 | phi156_005 | 1679 | 2638 | 319 | 37.14 | 9.15 | VSR homing endonuclease | Select seq YP_009195904.1  VSR homing endonuclease [*Staphylococcus* phage phiIPLA-RODI] | 1E-55 |
| 006 | phi156_006 | 2702 | 2995 | 97 | 10.89 | 7.92 | Phage terminase, large subunit | *Enterococcus* phage phiEF24C_NC_009904: putative large terminase; | 1.41E-57 |
| 007 | phi156_007 | 3286 | 4215 | 309 | 36.25 | 9 | VSR homing endonuclease | VSR homing endonuclease [*Staphylococcus* phage Quidividi] | 1E-51 |
| 008 | phi156_008 | 4370 | 5473 | 367 | 41.47 | 6.54 | Phage terminase, large subunit | *Enterococcus* phage phiEF24C_NC_009904: putative large terminase; | 0.00E+00 |
| 009 | phi156_009 | 5573 | 6361 | 262 | 29.54 | 7.58 |  | structural protein [*Staphylococcus* phage pSco-10 | 5E-26 |
| 010 | phi156_010 | 6466 | 7182 | 238 | 27.49 | 4.65 |  | hypothetical protein EFLK1_ORF198 [*Enterococcus* phage EFLK1]; | 1.62E-169 |
| 011 | phi156_011 | 7172 | 7516 | 114 | 12.87 | 6.58 |  | hypothetical protein EFLK1_ORF197 [*Enterococcus* phage EFLK1]; | 2.00E-77 |
| 012 | phi156_012 | 7610 | 8479 | 289 | 31.42 | 7.55 | Phage lysin, N-acetylmuramoyl-L-alanine amidase (EC 3.5.1.28) | *Enterococcus* phage phiEF24C_NC_009904: putative N-acetylmuramoyl-L-alanine amidase; | 0.00E+00 |
| 013 | phi156_013 | 8653 | 9291 | 212 | 23.11 | 4.42 | peptidoglycan-binding LysM | putative peptidoglycan-binding LysM [*Enterococcus* phage phiM1EF22] | 1.00E-149 |
| 014 | phi156_014 | 9435 | 9779 | 114 | 13.51 | 5.9 |  | *Enterococcus* phage phiEF24C_NC_009904: hypothetical protein; | 2.31E-79 |
| 015 | phi156_015 | 9794 | 11515 | 573 | 64.80 | 5.63 | Portal protein | *Enterococcus* phage phiEF24C_NC_009904: putative portal protein; | 0.00E+00 |
| 016 | phi156_016 | 11525 | 11638 | 37 | 3.94 | 7.95 |  | *Enterococcus* phage phiEF24C_NC_009904: hypothetical protein; | 2.51E-12 |
| 017 | phi156_017 | 11700 | 12416 | 238 | 26.73 | 5.37 | Putative prohead protease | *Enterococcus* phage phiEF24C_NC_009904: putative prohead protease; | 0.00E+00 |
| 018 | phi156_018 | 12423 | 13367 | 314 | 35.55 | 4.32 |  | *Enterococcus* phage phiEF24C_NC_009904: hypothetical protein; | 0.00E+00 |
| 019 | phi156_019 | 13508 | 14902 | 464 | 51.18 | 5.39 | Phage major capsid protein | *Enterococcus* phage phiEF24C_NC_009904: putative major capsid protein; | 0.00E+00 |
| 020 | phi156_020 | 15007 | 15270 | 87 | 9.66 | 6.74 |  | *Enterococcus* phage phiEF24C_NC_009904: hypothetical protein; | 2.00E-55 |
| 021 | phi156_021 | 15283 | 16182 | 299 | 34.02 | 4.89 |  | *Enterococcus* phage phiEF24C_NC_009904: hypothetical protein; | 0.00E+00 |
| 022 | phi156_022 | 16199 | 17068 | 289 | 32.55 | 6.48 | Phage capsid protein | *Enterococcus* phage phiEF24C_NC_009904: hypothetical protein; capsid protein; | 0.00E+00 |
| 023 | phi156_023 | 17061 | 17684 | 207 | 23.73 | 10.46 |  | *Enterococcus* phage phiEF24C_NC_009904: hypothetical protein; | 1.37E-149 |
| 024 | phi156_024 | 17688 | 18533 | 281 | 31.86 | 4.67 |  | *Enterococcus* phage phiEF24C_NC_009904: hypothetical protein; | 0.00E+00 |
| 025 | phi156_025 | 18533 | 18772 | 79 | 9.06 | 9.27 |  | *Enterococcus* phage phiEF24C_NC_009904: hypothetical protein; | 2.70E-32 |
| 026 | phi156_026 | 18776 | 20485 | 569 | 61.89 | 5.27 | Phage major tail sheath protein | *Enterococcus* phage phiEF24C_NC_009904: putative tail sheath protein; | 0.00E+00 |
| 027 | phi156_027 | 20546 | 20968 | 140 | 15.49 | 5.37 | Tail tube subunit | *Enterococcus* phage EFDG1; tail tube subunit; | 3E-96 |
| 028 | phi156_028 | 21060 | 22157 | 365 | 42.56 | 8.97 |  | *Enterococcus* phage EFLK1_NC_029026: hypothetical protein; | 0.00E+00 |
| 029 | phi156_029 | 22154 | 22300 | 48 | 5.81 | 4.78 |  | *Enterococcus* phage phiEF24C_NC_009904: hypothetical protein; | 2.44E-23 |
| 030 | phi156_030 | 22437 | 22910 | 157 | 18.29 | 4.87 | Tail assembly chaperone | Bacillus phage Troll; Tail assembly chaperone; | 1e-37 |
| 031 | phi156_031 | 22978 | 23553 | 191 | 22.42 | 4.37 | Putative RNA polymerase | *Enterococcus* phage phiEF24C_NC_009904: putative RNA polymerase; | 1.76E-135 |
| 032 | phi156_032 | 23631 | 27254 | 1207 | 128.44 | 8.45 | Putative Tail Lysin | *Enterococcus* phage EFLK1; putative tail lysin;  (endo-beta-N-acetylglucosamidase) | 0.00E+00 |
| 033 | phi156_033 | 27293 | 30478 | 1061 | 118.14 | 5.27 | Putative tail lysin | *Enterococcus* phage phiM1EF22; putative tail lysin;  (Cell wall associated hydrolase) | 0.00E+00 |
| 034 | phi156_034 | 30573 | 36041 | 1822 | 202.63 | 4.7 | Putative tail fibre | *Enterococcus* phage EFLK1_NC_029026: putative tail fibre; | 0.00E+00 |
| 035 | phi156_035 | 36264 | 38552 | 762 | 83.90 | 8.46 | Putative minor structural protein | *Enterococcus* phage ECP3_NC_027335: putative minor structural protein; | 0.00E+00 |
| 036 | phi156_036 | 38546 | 39271 | 241 | 27.93 | 4.38 |  | *Enterococcus* phage phiEF24C_NC_009904: hypothetical protein; | 2.48E-171 |
| 037 | phi156_037 | 39303 | 39449 | 48 | 5.66 | 4.93 |  | *Enterococcus* phage phiEF24C_NC_009904: hypothetical protein; | 4.70E-27 |
| 038 | phi156_038 | 39586 | 40275 | 229 | 25.55 | 9.11 | Structural protein | *Staphylococcus* phage vB_SauM_Romulus: structural protein; | 5e-44 |
| 039 | phi156_039 | 40279 | 40815 | 178 | 20.09 | 4.71 |  | *Enterococcus* phage phiM1EF22: hypothetical protein; | 1E-125 |
| 040 | phi156_040 | 40802 | 41506 | 234 | 26.36 | 4.69 | Putative baseplate protein | *Enterococcus* phage ECP3_NC_027335: putative baseplate; | 3E-173 |
| 041 | phi156_041 | 41522 | 42574 | 350 | 39.54 | 5.22 | Phage baseplate J protein | *Enterococcus* phage phiEF24C_NC_009904: putative structural protein; | 0.00E+00 |
| 042 | phi156_042 | 42593 | 43993 | 466 | 53.09 | 4.73 |  | *Enterococcus* phage phiEF24C_NC_009904: hypothetical protein; | 0.00E+00 |
| 043 | phi156_043 | 44099 | 44644 | 181 | 20.35 | 6.61 | Structural phage protein | *Enterococcus* phage phiEF24C_NC_009904: structural protein (Virion protein 3); ; Sequence ID: P85227.2 | 4E-131 |
| 044 | phi156_044 | 44659 | 48123 | 1154 | 128.92 | 4.99 | Putative phage tail adsoption protein | *Enterococcus* phage phiEF24C_NC_009904: putative adsorption associated tail protein; | 0.00E+00 |
| 045 | phi156_045 | 48200 | 48406 | 68 | 7.78 | 5.67 |  | *Enterococcus* phage phiEF24C_NC_009904: hypothetical protein; | 5.16E-41 |
| 046 | phi156_046 | 48661 | 51705 | 1014 | 114.60 | 5.92 | DNA helicase, phage-associated | *Enterococcus* phage phiM1EF22: putative helicase; | 0.00E+00 |
| 047 | phi156_047 | 51733 | 53361 | 542 | 62.91 | 8.56 | Putative transcriptional regulator | *Enterococcus* phage phiEF24C_NC_009904: putative transcriptional regulator; | 0.00E+00 |
| 048 | phi156_048 | 53394 | 55928 | 844 | 94.70 | 5.47 | Helicase/primase | *Enterococcus* phage EFP01: putative helicase; | 0.00E+00 |
| 049 | phi156_049 | 55928 | 56983 | 351 | 39.85 | 5.41 | Phage recombination exonuclease | *Enterococcus* phage phiEF24C_NC_009904: putative exonuclease; | 0.00E+00 |
| 050 | phi156_050 | 57099 | 58991 | 630 | 71.34 | 5.13 | Putative exonuclease | *Enterococcus* phage EFLK1_NC_029026: putative exonuclease; | 0.00E+00 |
| 051 | phi156_051 | 59000 | 59665 | 221 | 25.86 | 5.2 |  | *Enterococcus* phage phiEF17H_NC_029026.1:hypothetical protein; | 2E-157 |
| 052 | phi156_052 | 59666 | 60724 | 352 | 40.43 | 7.05 | DNA primase | *Enterococcus* phage phiEF24C_NC_009904: putative primase; | 0.00E+00 |
| 053 | phi156_053 | 60741 | 61373 | 210 | 24.24 | 5.08 | Putative cytidine deaminase | *Enterococcus* phage phiEF17H_NC_029026.1:putative cytidine deaminase; | 20E-136 |
| 054 | phi156_054 | 61399 | 62283 | 294 | 32.75 | 6.04 | Putative deoxyuridine 5'-triphosphate nucleotidohydrolase | *Listeria* phage LMTA-148: putative deoxyuridine 5'-triphosphate nucleotidohydrolase;  (Sequence ID: AID17402.1) | 1E-41 |
| 055 | phi156_055 | 62286 | 62516 | 76 | 8.95 | 8.66 | Putative dUTPase | *Enterococcus* phage EFLK1_NC_029026: putative dUTPase; | 4E-48 |
| 056 | phi156_056 | 62518 | 62826 | 102 | 12.09 | 9.12 |  | *Enterococcus* phage phiEF24C_NC_009904: hypothetical protein; | 3.09E-67 |
| 057 | phi156_057 | 62840 | 63124 | 94 | 11.07 | 4.51 | Putative phosphotransferase/anion transport protein | *Enterococcus* phage phiEF17H_NC_029026.1:putative phosphotransferase/anion transport protein; | 6.81E-67 |
| 058 | phi156_058 | 63117 | 63488 | 123 | 14.31 | 6.18 |  | *Enterococcus* phage phiEF24C_NC_009904: hypothetical protein; | 2.40E-83 |
| 059 | phi156_059 | 63508 | 64176 | 222 | 25.72 | 4.93 | Putative holliday junction resolvase | *Enterococcus* phage phiEF24C_NC_009904: putative resolvase; | 1.07E-165 |
| 060 | phi156_060 | 64178 | 64477 | 99 | 11.30 | 5.37 |  | *Enterococcus* phage phiEF24C_NC_009904: hypothetical protein; | 4.45E-64 |
| 061 | phi156_061 | 64483 | 64962 | 159 | 18.44 | 6.31 |  | *Enterococcus* phage phiEF24C_NC_009904: hypothetical protein; | 3.86E-113 |
| 062 | phi156_062 | 65055 | 65849 | 264 | 31.60 | 6.97 | Synaptonemal complex 1 domain containing protein | *Enterococcus* phage EFDG1: synaptonemal complex 1 domain containing protein; | 0.00E+00 |
| 063 | phi156_063 | 65842 | 66153 | 103 | 11.91 | 9.7 | Integration host factor | *Enterococcus* phage EFLK1_NC_029026: integration host factor; | 5E-68 |
| 064 | phi156_064 | 66243 | 68645 | 800 | 92.83 | 7.27 | Putative DNA polymerase I | *Enterococcus* phage EFLK1_NC_029026: putative DNA polymerase; | 0.00E+00 |
| 065 | phi156_065 | 68815 | 69477 | 220 | 25.63 | 9.3 | HTH homing endonuclease | *Bacillus* phage Grass: HTH homing endonuclease; | 2E-46 |
| 066 | phi156_066 | 69806 | 70330 | 174 | 20.46 | 5.07 | Putative DNA polymerase I | *Enterococcus* phage ECP3_NC_027335: putative DNA polymerase; | 1E-123 |
| 067 | phi156_067 | 70433 | 70975 | 180 | 21.52 | 5.23 |  | *Enterococcus* phage EFLK1_NC_029026: hypothetical protein; | 0.00E+00 |
| 068 | phi156_068 | 71032 | 72321 | 429 | 48.21 | 4.85 |  | *Enterococcus* phage phiEF24C_NC_009904: hypothetical protein; | 0.00E+00 |
| 069 | phi156_069 | 72406 | 73653 | 415 | 46.20 | 5.81 | Phage recombinase | *Enterococcus* phage phiEF24C_NC_009904: putative recombinase A; | 0.00E+00 |
| 070 | phi156_070 | 73707 | 74093 | 128 | 14.71 | 8.57 |  | *Enterococcus* phage phiEF24C_NC_009904: hypothetical protein; | 2.39E-91 |
| 071 | phi156_071 | 74086 | 74700 | 204 | 23.71 | 6.15 | Putative sigma factor | *Enterococcus* phage phiEF24C_NC_009904: putative sigma factor; | 2.99E-145 |
| 072 | phi156_072 | 74761 | 75036 | 91 | 10.22 | 6.18 | Phage holin protein | *Enterococcus* phage phiEF24C_NC_009904: putative holin; | 6.27E-62 |
| 073 | phi156_073 | 75084 | 76028 | 314 | 34.51 | 4.77 | with Ig-like domain | *Enterococcus* phage phiEF24C_NC_009904: putative Ig-like protein; | 0.00E+00 |
| 074 | phi156_074 | 76061 | 76504 | 147 | 16.62 | 4.58 | structural protein | *Enterococcus* phage phiEF24C_NC_009904: structural protein; | 1.76E-100 |
| 075 | phi156_075 | 76610 | 76906 | 98 | 11.38 | 4.66 |  | *Enterococcus* phage phiEF17H_NC_029026.1:hypothetical protein; | 1E-62 |
| 076 | phi156_076 | 76911 | 77864 | 317 | 35.88 | 5.83 |  | *Enterococcus* phage phiEF24C_NC_009904: hypothetical protein; | 0.00E+00 |
| 077 | phi156_077 | 77918 | 79201 | 427 | 48.81 | 5.72 | Putative DNA repair protein | *Enterococcus* phage EFDG1: putative DNA repair exonuclease; | 0.00E+00 |
| 078 | phi156_078 | 79213 | 79587 | 124 | 14.14 | 9.64 |  | *Enterococcus* phage phiEF24C_NC_009904: hypothetical protein; | 5.86E-82 |
| 079 | phi156_079 | 79626 | 80246 | 206 | 23.23 | 5.66 |  | *Enterococcus* phage phiEF24C_NC_009904: hypothetical protein; | 1.08E-147 |
| 080 | phi156_080 | 80246 | 80986 | 246 | 28.31 | 9.32 |  | *Enterococcus* phage phiEF24C_NC_009904: hypothetical protein; | 0.00E+00 |
| 081 | phi156_081 | 80976 | 81482 | 168 | 19.13 | 10.38 |  | *Enterococcus* phage phiEF24C_NC_009904: hypothetical protein; | 3.60E-115 |
| 082 | phi156_082 | 81496 | 82353 | 285 | 31.54 | 4.94 |  | *Enterococcus* phage phiEF24C_NC_009904: hypothetical protein; | 0.00E+00 |
| 083 | phi156_083 | 82457 | 83302 | 281 | 32.29 | 5.01 |  | *Enterococcus* phage EFLK1_NC_029026: hypothetical protein; | 0.00E+00 |
| 084 | phi156_084 | 83295 | 84923 | 542 | 62.98 | 8.86 |  | *Enterococcus* phage phiEF24C_NC_009904: hypothetical protein; | 0.00E+00 |
| 085 | phi156_085 | 85242 | 85931 | 229 | 26.29 | 4.86 |  | *Enterococcus* phage phiEF24C_NC_009904: hypothetical protein; | 4.00E-165 |
| 086 | phi156_086 | 85942 | 86409 | 155 | 18.17 | 4.78 |  | *Enterococcus* phage EFLK1_NC_029026: hypothetical protein; | 6.31E-109 |
| 087 | phi156_087 | 86506 | 88788 | 760 | 86.94 | 4.58 |  | *Enterococcus* phage EFLK1_NC_029026: hypothetical protein; | 0.00E+00 |
| 088 | phi156_088 | 88848 | 89054 | 68 | 7.46 | 5.43 |  | *Enterococcus* phage ECP3_NC_027335: hypothetical protein; | 2E-37 |
| 089 | phi156_089 | 89073 | 89579 | 168 | 18.50 | 5.57 |  | *Enterococcus* phage phiEF24C_NC_009904: hypothetical protein; | 3.82E-114 |
| 090 | phi156_090 | 89654 | 89824 | 56 | 6.69 | 4.41 |  | *Enterococcus* phage phiEF24C_NC_009904: hypothetical protein; | 1.31E-33 |
| 091 | phi156_091 | 89814 | 90086 | 90 | 10.32 | 4.41 |  | *Enterococcus* phage phiEF24C_NC_009904: hypothetical protein; | 1.47E-56 |
| 092 | phi156_092 | 90194 | 90445 | 83 | 9.60 | 9.17 | Putative transcriptional regulator | *Enterococcus* phage phiEF24C_NC_009904: putative transcriptional regulator; (XRE family) | 3E-52 |
| 093 | phi156_093 | 90457 | 90744 | 95 | 11.38 | 4.45 |  | *Enterococcus* phage phiEF24C_NC_009904: hypothetical protein; | 6.58E-61 |
| 094 | phi156_094 | 90747 | 91535 | 262 | 29.74 | 7.55 |  | *Enterococcus* phage phiEF24C_NC_009904: hypothetical protein; | 0.00E+00 |
| 095 | phi156_095 | 91617 | 92690 | 357 | 38.41 | 4.22 |  | *Enterococcus* phage phiEF24C_NC_009904: hypothetical protein; | 0.00E+00 |
| 096 | phi156_096 | 92816 | 93118 | 100 | 11.56 | 10.33 |  | *Enterococcus* phage EFLK1_NC_029026: hypothetical protein; | 3.05E-66 |
| 097 | phi156_097 | 93120 | 93422 | 100 | 11.71 | 3.96 |  | *Enterococcus* phage phiEF24C_NC_009904: hypothetical protein; | 2.26E-64 |
| 098 | phi156_098 | 93425 | 93709 | 94 | 10.33 | 9.48 |  | *Enterococcus* phage phiEF24C_NC_009904: hypothetical protein; | 4.18E-55 |
| 099 | phi156_099 | 93731 | 94081 | 116 | 13.55 | 4.03 |  | *Enterococcus* phage phiEF24C_NC_009904: hypothetical protein; | 1.65E-56 |
| 100 | phi156_100 | 94108 | 94485 | 125 | 14.49 | 8.86 |  | *Enterococcus* phage phiEF24C_NC_009904: hypothetical protein; | 9.74E-80 |
| 101 | phi156_101 | 94478 | 94708 | 76 | 8.87 | 5.12 |  | *Enterococcus* phage phiEF24C_NC_009904: hypothetical protein; | 1.46E-44 |
| 102 | phi156_102 | 94712 | 95086 | 124 | 14.23 | 4.58 |  | *Enterococcus* phage phiEF24C_NC_009904: hypothetical protein; | 2.58E-80 |
| 103 | phi156_103 | 95083 | 95265 | 60 | 7.15 | 4.78 |  | *Enterococcus* phage phiEF17H_NC_029026.1:hypothetical protein; | 3E-14 |
| 104 | phi156_104 | 95266 | 95895 | 209 | 23.79 | 5.18 |  | *Enterococcus* phage phiEF24C_NC_009904: hypothetical protein; | 1.53E-149 |
| 105 | phi156_105 | 95974 | 96216 | 80 | 9.57 | 5.66 |  | *Enterococcus* phage phiEF24C_NC_009904: hypothetical protein; | 5.60E-50 |
| 106 | phi156_106 | 96233 | 96373 | 46 | 5.53 | 9.33 |  | *Enterococcus* phage phiEF24C_NC_009904: hypothetical protein; | 6.68E-24 |
| 107 | phi156_107 | 96386 | 96718 | 110 | 12.92 | 5.43 |  | *Enterococcus* phage EFLK1_NC_029026: hypothetical protein; | 5E-75 |
| 108 | phi156_108 | 96783 | 97439 | 218 | 24.61 | 5.07 |  | *Enterococcus* phage phiEF24C_NC_009904: hypothetical protein; | 1.07E-144 |
| 109 | phi156_109 | 97436 | 97879 | 147 | 16.70 | 5.52 |  | *Enterococcus* phage phiEF24C_NC_009904: hypothetical protein; | 7.69E-100 |
| 110 | phi156_110 | 97932 | 98492 | 186 | 20.97 | 6.83 |  | *Enterococcus* phage EFP01: hypothetical protein; | 1E-113 |
| 111 | phi156_111 | 98710 | 98946 | 78 | 9.16 | 8.03 |  | *Enterococcus* phage EFDG1: hypothetical protein; | 4E-41 |
| 112 | phi156_112 | 99008 | 99196 | 62 | 6.86 | 5.19 | XRE family transcriptional regulator | *Enterococcus*: XRE family transcriptional regulator; (HTH motif protein) | 2E-31 |
| 113 | phi156_113 | 99190 | 99303 | 37 | 4.18 | 4.44 |  | *Enterococcus* phage EFDG1: hypothetical protein; | 5E-16 |
| 114 | phi156_114 | 99410 | 99652 | 80 | 9.17 | 7.88 |  | *Enterococcus* phage SANTOR1: hypothetical protein; | 7E-51 |
| 115 | phi156_115 | 99826 | 100275 | 149 | 17.38 | 4.22 |  | *Enterococcus* phage EFP01: hypothetical protein; | 8E-92 |
| 116 | phi156_116 | 100331 | 100693 | 120 | 13.37 | 12.78 | Putative pyruvate kinase | *Kosakonia oryzae*: pyruvate kinase; | 6E-27 |
| 117 | phi156_117 | 100894 | 101193 | 99 | 11.15 | 9.03 |  | *Enterococcus* phage phiEF24C_NC_009904: hypothetical protein; | 2.85E-55 |
| 118 | phi156_118 | 101271 | 101594 | 107 | 12.42 | 4.87 |  | *Enterococcus* phage phiEF24C_NC_009904: hypothetical protein; | 1.08E-63 |
| 119 | phi156_119 | 101691 | 101831 | 46 | 5.49 | 10.2 |  | *Enterococcus* phage phiEF24C_NC_009904: hypothetical protein; | 6.94E-20 |
| 120 | phi156_120 | 101890 | 102096 | 68 | 7.51 | 9.18 |  | *Enterococcus* phage EFLK1_NC_029026: hypothetical protein_ORF081; | 4E-38 |
| 121 | phi156_121 | 102173 | 102424 | 83 | 9.62 | 8.97 |  | *Enterococcus* phage phiEF24C_NC_009904: hypothetical protein; | 1.74E-51 |
| 122 | phi156_122 | 102491 | 102721 | 76 | 9.02 | 3.76 |  | *Enterococcus* phage phiEF24C_NC_009904: hypothetical protein; | 2.69E-42 |
| 123 | phi156_123 | 102771 | 102977 | 68 | 7.96 | 6.23 |  | *Enterococcus* phage phiEF24C_NC_009904: hypothetical protein; | 6.78E-40 |
| 124 | phi156_124 | 102996 | 103166 | 56 | 6.84 | 10.13 |  | *Enterococcus* phage phiEF24C_NC_009904: hypothetical protein; | 4.78E-30 |
| 125 | phi156_125 | 103310 | 103639 | 109 | 12.67 | 4.26 |  | *Streptococcus* phage SPQS1]_NC_021868: hypothetical protein; | 8E-54 |
| 126 | phi156_126 | 103721 | 104017 | 98 | 11.33 | 8.87 |  | *Enterococcus* phage phiEF24C_NC_009904: hypothetical protein; | 5.05E-67 |
| 127 | phi156_127 | 104096 | 104308 | 70 | 8.27 | 6.58 |  | *Enterococcus* phage phiEF24C_NC_009904: hypothetical protein; | 4.16E-41 |
| 128 | phi156_128 | 104305 | 104610 | 101 | 11.86 | 6.56 |  | *Enterococcus* phage phiEF17H_NC_029026.1:hypothetical protein PHIEF17H_1280; | 4E-65 |
| 129 | phi156_129 | 104598 | 104789 | 63 | 7.35 | 4.7 |  | *Enterococcus* phage phiEF24C_NC_009904: hypothetical protein; | 4.42E-35 |
| 130 | phi156_130 | 104801 | 105160 | 119 | 13.95 | 9.99 |  | *Enterococcus* phage phiEF24C_NC_009904: hypothetical protein; | 7.09E-74 |
| 131 | phi156_131 | 106037 | 106219 | 60 | 7.28 | 4.06 |  | *Enterococcus* phage VD13_NC_024212: hypothetical protein X878_0078; | 7E-23 |
| 132 | phi156_132 | 106301 | 106642 | 113 | 13.04 | 4.28 |  | hypothetical; |  |
| 133 | phi156_133 | 106700 | 106834 | 44 | 5.14 | 4.55 |  | *Enterococcus* phage phiSHEF2: hypothetical protein phiSHEF2_59; | 3E-19 |
| 134 | phi156_134 | 106977 | 107204 | 75 | 8.80 | 5.14 |  | *Enterococcus* phage ECP3_NC_027335: hypothetical protein; | 7E-45 |
| 135 | phi156_135 | 107288 | 107563 | 91 | 10.61 | 7.81 |  | *Enterococcus* phage EF-P29: hypothetical protein EFP29_02; | 4E-52 |
| 136 | phi156_136 | 107611 | 107790 | 59 | 6.90 | 9.61 |  | *Enterococcus* phage phiEF24C_NC_009904: hypothetical protein; | 1.01E-35 |
| 137 | phi156_137 | 107744 | 107899 | 51 | 5.87 | 9.52 |  | *Enterococcus* phage ECP3_NC_027335: hypothetical protein; | 0.005 |
| 138 | phi156_138 | 107982 | 108125 | 47 | 5.74 | 3.96 |  | *Enterococcus* phage ECP3_NC_027335: hypothetical protein; | 7E-23 |
| 139 | phi156_139 | 108405 | 108262 | 47 | 5.43 | 9.68 |  | *Enterococcus* phage phiEF24C_NC_009904: hypothetical protein; | 1.76E-16 |
| 140 | phi156_140 | 109032 | 108712 | 106 | 12.26 | 5.15 |  | *Enterococcus* phage phiEF24C_NC_009904: hypothetical protein; | 1.25E-68 |
| 141 | phi156_141 | 109397 | 109086 | 103 | 11.27 | 4.93 |  | *Enterococcus* phage phiEF17H_NC_029026.1:hypothetical protein PHIEF17H_1400; | 4.57E-64 |
| 142 | phi156_142 | 109658 | 109425 | 77 | 8.60 | 9.52 |  | *Enterococcus* phage phiEF24C_NC_009904: hypothetical protein; | 3.41E-06 |
| 143 | phi156_143 | 109853 | 109659 | 64 | 7.36 | 6 |  | *Enterococcus* phage phiEF24C_NC_009904: hypothetical protein; | 3.43E-36 |
| 144 | phi156_144 | 110374 | 110183 | 63 | 6.99 | 6.53 |  | *Enterococcus* phage phiEF24C_NC_009904: hypothetical protein; | 3.98E-37 |
| 145 | phi156_145 | 110636 | 110418 | 72 | 8.15 | 3.82 |  | *Enterococcus* phage phiEF24C_NC_009904: hypothetical protein; | 8.49E-44 |
| 146 | phi156_146 | 110901 | 110650 | 83 | 9.84 | 4.94 |  | *Enterococcus* phage EFLK1_NC_029026: hypothetical protein EFLK1_ORF050; | 2E-55 |
| 147 | phi156_147 | 111282 | 110902 | 126 | 14.40 | 4.19 |  | *Enterococcus* phage ECP3_NC_027335: hypothetical protein; | 7E-60 |
| 148 | phi156_148 | 111707 | 111510 | 65 | 7.45 | 4.54 |  | *Enterococcus* phage phiEF24C_NC_009904: hypothetical protein; | 2.18E-41 |
| 149 | phi156_149 | 112222 | 111707 | 171 | 19.59 | 4.57 |  | *Enterococcus* phage EFLK1_NC_029026: hypothetical protein EFLK1_ORF045; | 2E-122 |
| 150 | phi156_150 | 112483 | 112235 | 82 | 9.37 | 4.82 |  | *Enterococcus* phage phiEF24C_NC_009904: hypothetical protein; | 4.68E-50 |
| 151 | phi156_151 | 113038 | 112496 | 180 | 21.16 | 5.79 |  | *Enterococcus* phage phiEF24C_NC_009904: hypothetical protein; | 1.54E-108 |
| 152 | phi156_152 | 113427 | 113035 | 130 | 15.65 | 5.1 |  | *Enterococcus* phage phiEF24C_NC_009904: hypothetical protein; | 1.19E-84 |
| 153 | phi156_153 | 113780 | 113424 | 118 | 13.77 | 4.67 |  | *Enterococcus* phage phiEF24C_NC_009904: hypothetical protein; | 8.89E-80 |
| 154 | phi156_154 | 113954 | 113784 | 56 | 6.37 | 3.73 |  | *Enterococcus* phage phiEF24C_NC_009904: hypothetical protein; | 2.14E-33 |
| 155 | phi156_155 | 114484 | 113942 | 180 | 20.80 | 8.4 |  | *Enterococcus* phage EFLK1_NC_029026: hypothetical protein EFLK1_ORF039; | 2E-128 |
| 156 | phi156_156 | 114951 | 114487 | 154 | 18.06 | 8.81 |  | *Enterococcus* phage EFLK1_NC_029026: hypothetical protein EFLK1_ORF038; | 5E-111 |
| 157 | phi156_157 | 115418 | 114948 | 156 | 18.23 | 8.39 |  | *Enterococcus* phage EFLK1_NC_029026: hypothetical protein EFLK1_ORF037; | 8E-109 |
| 158 | phi156_158 | 115634 | 115434 | 66 | 7.86 | 6.24 |  | *Enterococcus* phage EFLK1_NC_029026: hypothetical protein EFLK1_ORF036; | 2E-39 |
| 159 | phi156_159 | 116112 | 115747 | 121 | 13.90 | 5.51 |  | *Enterococcus* phage EFLK1_NC_029026: hypothetical protein EFLK1_ORF034; | 7.05E-84 |
| 160 | phi156_160 | 116288 | 116109 | 59 | 6.75 | 3.63 |  | *Enterococcus* phage EFLK1_NC_029026: hypothetical protein EFLK1_ORF033; | 8E-32 |
| 161 | phi156_161 | 116820 | 116302 | 172 | 19.92 | 9.62 |  | *Enterococcus* phage EFLK1_NC_029026: hypothetical protein EFLK1_ORF032; | 3E-125 |
| 162 | phi156_162 | 117294 | 116821 | 157 | 18.23 | 6.44 |  | *Enterococcus* phage EFLK1_NC_029026: hypothetical protein EFLK1_ORF031; | 7.50E-92 |
| 163 | phi156_163 | 117998 | 117291 | 235 | 26.30 | 5.26 |  | *Enterococcus* phage EFLK1_NC_029026: hypothetical protein EFLK1_ORF030; | 6E-174 |
| 164 | phi156_164 | 118467 | 118027 | 146 | 16.86 | 5.02 |  | *Enterococcus* phage phiEF24C_NC_009904: hypothetical protein; | 8.46E-102 |
| 165 | phi156_165 | 118705 | 118517 | 62 | 7.66 | 5.17 |  | *Enterococcus* phage phiEF24C_NC_009904: hypothetical protein; | 5.62E-35 |
| 166 | phi156_166 | 119163 | 118702 | 153 | 16.79 | 4.05 |  | *Enterococcus* phage phiM1EF22: hypothetical protein PHIM1EF22_1620 | 9E-105 |
| 167 | phi156_167 | 119353 | 119177 | 58 | 6.69 | 4.09 |  | *Enterococcus* phage phiEF24C_NC_009904: hypothetical protein; | 7.17E-25 |
| 168 | phi156_168 | 119544 | 119350 | 64 | 7.72 | 6.26 |  | *Enterococcus* phage phiEF24C_NC_009904: hypothetical protein; | 2.67E-32 |
| 169 | phi156_169 | 119978 | 119541 | 145 | 17.53 | 9.84 |  | *Enterococcus* phage phiEF24C_NC_009904: hypothetical protein; | 2.10E-96 |
| 170 | phi156_170 | 120810 | 120070 | 246 | 27.73 | 5.06 | Serine/threonine protein phosphatase (EC 3.1.3.16) | *Enterococcus* phage phiEF24C_NC_009904: putative serine/threonine protein phosphatase; | 2.05E-177 |
| 171 | phi156_171 | 121286 | 120807 | 159 | 18.12 | 9.4 |  | *Enterococcus* phage phiEF24C_NC_009904: hypothetical protein; | 4.63E-107 |
| 172 | phi156_172 | 121541 | 121290 | 83 | 10.27 | 9.82 |  | *Enterococcus* phage phiEF24C_NC_009904: hypothetical protein; | 9.79E-53 |
| 173 | phi156_173 | 122149 | 121538 | 203 | 23.54 | 6.87 | putative metallo-dependent phosphatase1 | *Enterococcus* phage EFLK1_NC_029026: putative metallo-dependent phosphatase1 | 4E-144 |
| 174 | phi156_174 | 122512 | 122162 | 116 | 13.44 | 8.67 |  | *Enterococcus* phage phiEF17H_NC_029026.1:hypothetical protein PHIEF17H_1720; | 3E-77 |
| 175 | phi156_175 | 122625 | 122509 | 38 | 4.22 | 3.84 |  | *Enterococcus* phage phiEF24C_NC_009904: hypothetical protein; | 1.00E-20 |
| 176 | phi156_176 | 122851 | 122639 | 70 | 7.96 | 4.53 |  | *Enterococcus* phage phiEF24C_NC_009904: hypothetical protein; | 1.22E-42 |
| 177 | phi156_177 | 123350 | 122937 | 137 | 15.97 | 4.89 |  | *Enterococcus* phage phiEF24C_NC_009904: hypothetical protein; | 5.77E-99 |
| 178 | phi156_178 | 123738 | 123355 | 127 | 14.52 | 4.81 |  | *Enterococcus* phage phiEF24C_NC_009904: hypothetical protein; | 1.30E-85 |
| 179 | phi156_179 | 124015 | 123740 | 91 | 11.07 | 4.82 |  | *Enterococcus* phage EFLK1_NC_029026: hypothetical protein EFLK1_ORF014; | 3E-60 |
| 180 | phi156_180 | 124205 | 123978 | 75 | 9.00 | 4.94 |  | *Enterococcus* phage EFLK1_NC_029026: hypothetical protein EFLK1_ORF013; | 5E-47 |
| 181 | phi156_181 | 124467 | 124294 | 57 | 6.57 | 9.22 |  | *Enterococcus* phage phiEF24C_NC_009904: hypothetical protein; | 6.83E-33 |
| 182 | phi156_182 | 124801 | 124457 | 114 | 13.39 | 4.96 |  | *Enterococcus* phage phiEF17H_NC_029026.1:hypothetical protein PHIEF17H_1790; | 5E-73 |
| 183 | phi156_183 | 125043 | 124801 | 80 | 9.14 | 4.58 |  | *Enterococcus* phage phiEF24C_NC_009904: hypothetical protein; | 6.69E-52 |
| 184 | phi156_184 | 125290 | 125036 | 84 | 9.97 | 5.03 |  | *Enterococcus* phage phiEF24C_NC_009904: hypothetical protein; | 9.99E-54 |
| 185 | phi156_185 | 125636 | 125301 | 111 | 12.84 | 4.57 |  | *Enterococcus* phage phiEF24C_NC_009904: hypothetical protein; | 6.19E-77 |
| 186 | phi156_186 | 126048 | 125611 | 145 | 17.27 | 5.69 |  | *Enterococcus* phage phiEF24C_NC_009904: hypothetical protein; | 3.34E-99 |
| 187 | phi156_187 | 126525 | 126064 | 153 | 17.40 | 4.84 |  | *Enterococcus* phage EFLK1_NC_029026: hypothetical protein EFLK1_ORF006; | 3.26E-108 |
| 188 | phi156_188 | 126745 | 126518 | 75 | 8.45 | 5 |  | *Enterococcus* phage EFLK1_NC_029026: hypothetical protein EFLK1_ORF005; | 1E-44 |
| 189 | phi156_189 | 127164 | 126733 | 143 | 16.69 | 4.66 |  | *Enterococcus* phage phiEF24C_NC_009904: hypothetical protein; | 3.26E-102 |
| 190 | phi156_190 | 127400 | 127167 | 77 | 8.78 | 3.85 |  | *Enterococcus* phage phiEF24C_NC_009904: hypothetical protein; | 1.39E-45 |
| 191 | phi156_191 | 127828 | 127397 | 143 | 16.47 | 4.74 |  | *Enterococcus* phage phiEF24C_NC_009904: hypothetical protein; | 9.23E-96 |
| 192 | phi156_192 | 128254 | 127832 | 140 | 16.09 | 8.74 | DNA methyltransferase | *Enterococcus* faecalis: DNA (cytosine-5-)-methyltransferase | 2E-81 |
| 193 | phi156_193 | 128555 | 128325 | 76 | 8.86 | 6.57 | DNA methyltransferase | *Enterococcus* faecalis: DNA (cytosine-5-)-methyltransferase | 6E-45 |
| 194 | phi156_194 | 129006 | 128677 | 109 | 12.20 | 4.14 | Ribonucleotide reductase class 1b (aerobic), beta subunit | *Enterococcus* phage phiEF24C_NC_009904: putative ribonucleotide reductase; | 5.99E-73 |
| 195 | phi156_195 | 129231 | 129085 | 48 | 5.47 | 8.8 | Putative protein | *Enterococcus* phage phiEF24C_NC_009904: hypothetical protein; .2; | 1.00E-07 |
| 196 | phi156_196 | 130003 | 129374 | 209 | 24.35 | 8.82 | HTH homing endonuclease | PHAGE_Lactoc_WRP3_NC_027341: HTH homing endonuclease; | 3.16E-35 |
| 197 | phi156_197 | 130705 | 130118 | 195 | 22.43 | 4.83 | Ribonucleotide reductase of class 1b (aerobic), beta subunit (EC 1.17.4.1) | *Enterococcus* phage phiEF24C_NC_009904: putative ribonucleotide reductase; | 1.49E-140 |
| 198 | phi156_198 | 132868 | 130718 | 716 | 80.72 | 5.15 | Ribonucleotide reductase class 1b (aerobic), alpha subunit (EC 1.17.4.1) | *Enterococcus* phage phiEF24C_NC_009904: putative ribonucleotide reductase; | 0.00E+00 |
| 199 | phi156_199 | 133113 | 132871 | 80 | 8.99 | 5.74 | Glutaredoxin-like protein NrdH, required for reduction of Ribonucleotide reductase class Ib | *Enterococcus* phage phiEF24C_NC_009904: putative ribonucleotide reductase; | 1.85E-53 |
| 200 | phi156_200 | 133509 | 133243 | 88 | 9.91 | 4.73 |  | *Enterococcus* phage phiEF24C_NC_009904: hypothetical protein; | 1.27E-54 |
| 201 | phi156_201 | 133820 | 133515 | 101 | 11.90 | 8.65 |  | *Enterococcus* phage phiEF24C_NC_009904: hypothetical protein; | 2.23e-65 |
| 202 | phi156_202 | 134141 | 133911 | 76 | 8.93 | 8.01 | Putative phage transcriptional regulator protein | *Enterococcus* phage phiEF24C_NC_009904: putative transcriptional regulator; (XRE-family transcriptional regulator) | 1e-45 |
| 203 | phi156_203 | 134429 | 134205 | 74 | 8.44 | 9.81 |  | *Enterococcus* phage phiEF24C_NC_009904: hypothetical protein; | 2.10e-47 |
| 204 | phi156_204 | 134824 | 134540 | 94 | 11.07 | 5.03 |  | *Enterococcus* phage phiEF17H_NC_029026.1:hypothetical protein PHIEF17H_2010; | 6e-62 |
|  |  | 134947 | 134876 |  |  |  | tRNA-Thr-TGT | tRNA-Thr-TGT |  |
| 205 | phi156_205 | 136064 | 134973 | 363 | 41.82 | 4.96 | with ATP binding motive | *Enterococcus* phage phiEF24C_NC_009904: hypothetical protein; | 0.00E+00 |
|  |  | 136508 | 136435 |  |  |  | tRNA-Met-CAT | tRNA-Met-CAT |  |
|  |  | 136640 | 136567 |  |  |  | tRNA-Ile-GAT | tRNA-Ile-GAT |  |
|  |  | 137663 | 137590 |  |  |  | tRNA-Arg-TCT | tRNA-Arg-TCT |  |
|  |  | 138220 | 138149 |  |  |  | tRNA-Trp-CCA | tRNA-Trp-CCA |  |
|  |  | 138543 | 138471 |  |  |  | tRNA-Pseudo-GTC | tRNA-Pseudo-GTC |  |
| 206 | phi156_206 | 139347 | 139213 | 44 | 4.97 | 8.93 |  | *Enterococcus* phage phiEF24C_NC_009904: hypothetical protein; | 3E-20 |
| 207 | phi156_207 | 140360 | 140022 | 112 | 12.66 | 4.99 |  | *Enterococcus* phage phiEF17H_NC_029026.1: putative structural protein | 4E-63 |
| 208 | phi156_208 | 140768 | 140424 | 114 | 13.27 | 4.91 |  | *Enterococcus* phage phiEF24C_NC_009904: hypothetical protein; | 8E-76 |
| 209 | phi156_209 | 141063 | 140803 | 86 | 9.93 | 7.7 |  | *Enterococcus* phage phiEF24C_NC_009904: hypothetical protein; | 2E-53 |
